# Predicting atrial fibrillation recurrence after radiofrequency ablation using the AIFV system: a prospective observational study

**DOI:** 10.1186/s12872-026-05860-3

**Published:** 2026-05-09

**Authors:** Yingrong Xin, Jingchao Li, Huihui Song, Luqian Cui, Haijia Yu, Yingjie Chu, Shujuan Dong

**Affiliations:** 1 Department of Cardiology, Zhengzhou University People’s Hospital, Henan Provincial People’s Hospital, Zhengzhou, 450003 China; 2 Department of Cardiology, Henan Provincial People’s Hospital, Zhengzhou University, Zhengzhou, 450003 China; 3 Department of Cardiac Care Unit, Henan Provincial People’s Hospital, Zhengzhou University, Zhengzhou, 450003 China

**Keywords:** Atrial fibrillation, Catheter ablation, Pulmonary vein isolation, AIFV system, Vistag scores

## Abstract

**Background:**

Pulmonary vein isolation (PVI) has become the cornerstone of atrial fibrillation (AF) treatment. Nevertheless, the efficacy of radiofrequency ablation remains limited by a substantial rate of recurrence. The selection of anesthesia, the surgeon's operative experience and medical expertise, as well as the ablation surgery strategy, all have an impact on the recurrence after atrial fibrillation surgery. Our research aims to explore a novel method for predicting atrial fibrillation (AF) recurrence.

**Methods:**

This study enrolled 148 patients with atrial fibrillation (AF) undergoing first-time radiofrequency ablation. Based on the data from the AIFV system (an artificial intelligence-based ablation quality analysis system that generates structured Vistag scores from CARTO3 mapping data), patients were divided into two groups: the high-score group(Vistag analysis scores ≥ V5) and the low-score group(scores < V5). Baseline characteristics, intraoperative parameters, and AIFV system-related data were collected. Multivariate logistic regression analysis was performed to identify factors associated with atrial fibrillation (AF) recurrence.

**Results:**

Among the 148 enrolled patients, 84 were categorized into the high-score group and 64 into the low-score group. Patients in the low-score group had significantly higher Left Ventricular End-Diastolic Volume (LVEDV), Left Ventricular Ejection Fraction (LVEF), and Left Ventricular End-Systolic Volume (LVESV), as well as a higher prevalence of diabetes (29.5% vs. 9.5%, *p =* 0.002). During a minimum follow-up of 365 days, 11 patients (13.1%) in the high-score group experienced recurrence of atrial arrhythmia, compared to 19 patients (29.7%) in the low-score group. Kaplan–Meier analysis revealed a significantly higher atrial arrhythmia-free survival rate in the high-score group (log-rank test, *P =* 0.012). Multivariate logistic regression analysis revealed that being in the high-score group was an independent protective factor against AF recurrence (OR = 0.383, 95% CI 0.160–0.912, *P =* 0.030).

**Conclusions:**

The AIFV system was used to evaluate six procedural parameters in each atrial fibrillation procedure, which could play a crucial role in predicting the recurrence of atrial fibrillation.

## Background

Atrial Fibrillation (AF) is a highly prevalent and clinically significant arrhythmia. Its prevalence has been gradually increasing in recent years and has become a growing global health concern [1–3]. According to epidemiological data,the incidence of AF has nearly doubled over the past several decades, imposing a heavy burden on the global healthcare system [2, 4, 5]. AF not only leads to uncomfortable symptoms such as palpitations, chest tightness, and fatigue, which significantly impair patients' quality of life [6, 7], but also markedly increases the risk of complications, including stroke [8], heart failure, and dementia [9], thereby contributing to a higher mortality rate among affected individuals [10]. Statistical data indicate that AF patients not receiving anticoagulant therapy have a 3- to fivefold increased risk of stroke compared to the general population, with persistently elevated post-stroke mortality and recurrence rates [11].

Currently, substantial research has been conducted on catheter ablation as a primary strategy for rhythm control in patients with AF [12–14]. Radiofrequency energy generates heat through tissue impedance, leading to myocardial cell degeneration and necrosis. Continuous ablation of the cardiac wall disrupts the electrical connectivity of abnormal myocardial tissue [15], thereby maintaining sinus rhythm. PVI is the cornerstone of AF catheter ablation [14, 16, 17]. However, research shows that among 31 study cohorts with a total of 2,800 patients, the single-procedure success rate of catheter ablation for atrial fibrillation was reported to be 57% [18]. Furthermore, experimental studies have demonstrated that, by the 12-month follow-up period, 36.4% of patients who underwent atrial fibrillation ablation experienced their first recurrence of atrial fibrillation [19]. The type of atrial fibrillation and the selected anesthesia approach can influence the recurrence rate following atrial fibrillation surgery [20, 21]. Additionally, recent studies have demonstrated that the combination of linear ablation with ethanol infusion of the vein of Marshall (EIVOM), when added to PVI, significantly improves freedom from atrial arrhythmias within 12 months compared with PVI alone [22].

PVI is a core step in atrial fibrillation (AF) ablation, aiming to cause transmural, continuous, and permanent cellular damage without damaging surrounding structures. The quality of PVI serves as a critical determinant of the overall efficacy of AF ablation procedures, having a direct impact on the clinical outcomes of patients undergoing AF ablation. Currently, there is a paucity of quantitative comparative data regarding the procedural quality of radiofrequency catheter ablation (RFCA) in the treatment of atrial fibrillation. Our study aims to investigate a novel assessment system for predicting the recurrence of atrial fibrillation (AF) following ablation procedures. During each AF ablation procedure, six procedural parameters were assessed using the AIFV system, which can predict the recurrence of AF.

## Methods

### Study design

This is a Single-center, real-world, prospective analysis study. The study cohort consisted of 148 patients with atrial fibrillation undergoing their first radiofrequency catheter ablation (RFCA) procedure during January 2023 to January 2024. Patients were classified based on the relevant data of the postoperative AIFV system (an artificial intelligence-based ablation quality analysis system that generates structured Vistag scores from CARTO3 mapping data): The Vistag analysis results with a total score ≥ V5 are classified as the high—score group, and those with a total score < V5 are classified as the low—score group. Collect baseline characteristics,intraoperative data, and the AIFV system-related data. Conduct a multivariate logistic analysis to identify the factors influencing the recurrence of atrial fibrillation.

### Study population

**Fig. 1:**
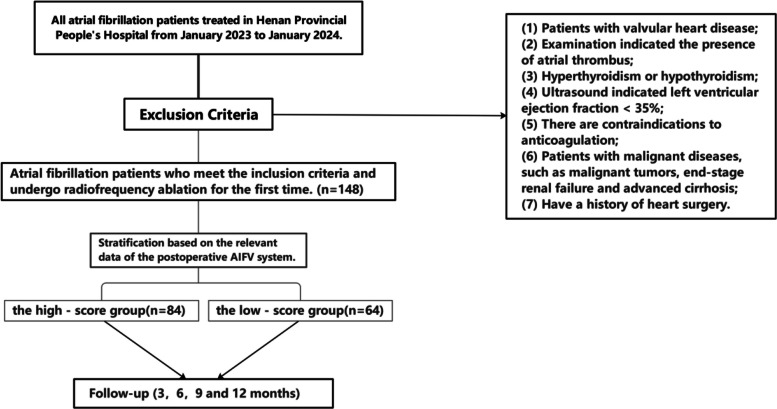
The overall process of this study

A consecutive cohort of patients with atrial fibrillation who underwent their initial radiofrequency ablation procedure at Henan Provincial People's Hospital between January 2023 and January 2024 was enrolled in the study, provided they met the predefined inclusion and exclusion criteria. The exclusion criteria were as following: (1) Patients with valvular heart disease (2); Examination indicated the presence of atrial thrombus; (3) Hyperthyroidism or hypothyroidism; (4) Ultrasound indicated left ventricular ejection fraction < 35%;(5) There are contraindications to anticoagulation; (6) Patients with malignant diseases, such as malignant tumors, end-stage renal failure and advanced cirrhosis; (7) Have a history of heart surgery. The exclusion criteria are illustrated in Fig. 1.

#### Diagnostic criteria

According to the《2024 ESC Guidelines for the management of Atrial Fibrillation developed in collaboration with the European Association for Cardio-Thoracic Surgery(EACTS)》 [23], the diagnostic criteria for atrial fibrillation (AF) are as follows: Atrial fibrillation is defined as the loss of regularity and order in atrial electrical activity, replaced by rapid and disordered fibrillation waves (f-waves), resulting in ineffective atrial contractions. Its electrocardiogram features include: (1) disappearance of P waves, replaced by irregular f-waves (with varying morphology, amplitude, and intervals); (2) absolutely irregular RR intervals; (3) inconsistent intervals of continuous atrial electrical activity (f-wave cycles) with a duration < 200 ms, corresponding to an atrial rate of 350–600 beats per minute; (4) occasional wide QRS complexes with abnormal morphology, accompanied by intraventricular conduction disorders.

#### Radiofrequency ablation strategy

1. Following circumferential pulmonary vein isolation, all patients underwent evaluation with the Pentaray electrode catheter and pacing ablation catheters within the pulmonary vein ablation ring to confirm the presence of bidirectional electrical conduction block between the bilateral pulmonary veins and the left atrium, signifying successful bilateral pulmonary vein isolation. In instances where pulmonary vein potentials were detected, targeted focal ablations were promptly delivered at the sites of potential recurrence to achieve complete electrical disconnection.
2. Electrophysiologists can formulate personalized ablation strategies according to intraoperative conditions, which may involve supplementary linear ablation at the mitral isthmus, tricuspid isthmus ablation, top-line ablation, Marshall vein alcohol ablation, and other relevant procedures.
3. If the electrocardiogram monitoring after circulatory pulmonary vein isolation still indicated atrial fibrillation, external direct-current cardioversion (DCCV) was performed to restore sinus rhythm.
4. Left atrial electroanatomical mapping using a matrix system was conducted in all enrolled patients diagnosed with atrial fibrillation (AF).
5. After completing all the ablation lines in the left atrium, a thorough examination of bilateral pulmonary veins and bidirectional conduction block at the circumpulmonary vein ablation ring, and additional ablation lines was conducted.

### Evaluation indicators of the AIFV system

**Fig. 2:**
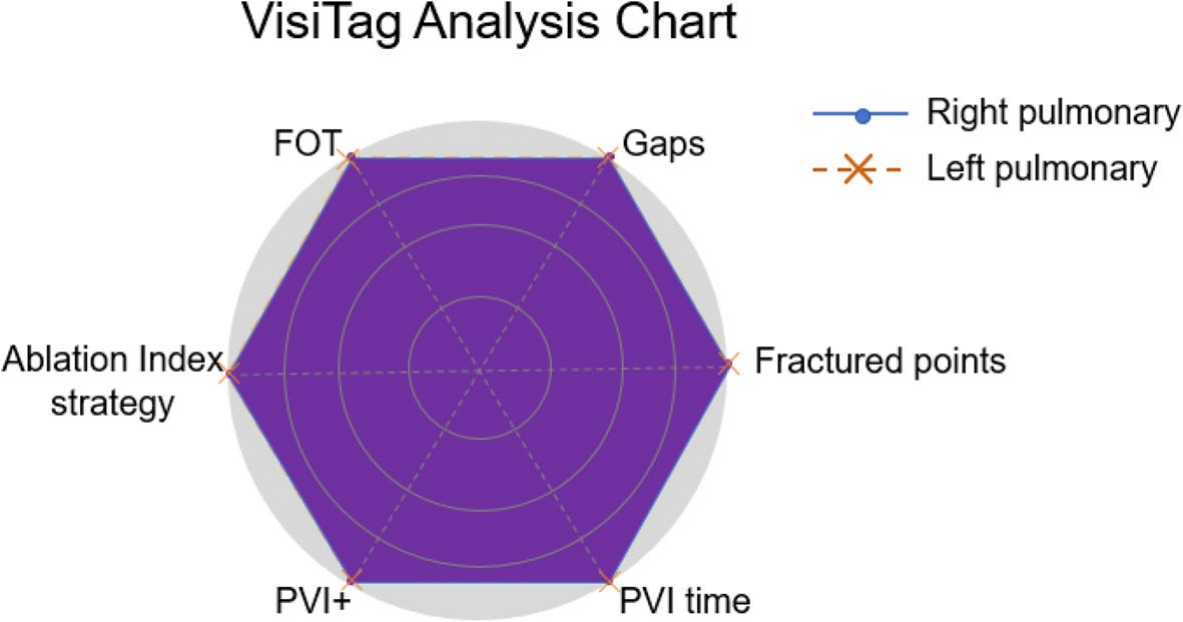
The Vistag analysis chart in the AIFV system includes fractured points, Gaps, FOT(force-over-time), PVI time, Ablation Index Strategy, and PVI +

The AIFV system is designed to assess the quality of radiofrequency ablation procedures in patients with atrial fibrillation. The Vistag analysis report integrates quantitative evaluation parameters for point-by-point mapping, linear continuity, and transmural lesion formation during the ablation process, including fractured points, Gaps, Ablation Index strategy, FOT (force-over-time), PVI time, and PVI + (Fig. 2).

### The Vistag score construction 

**Table 1:** The Vistag score construction and definition of the parameter

Parameter	Definition	Optimal Criterion	Score Contribution	
			Left pulmonary veins	Right pulmonary veins
Fractured points	Refers to the number of Tag points ≥ 6 within a range of 6 mm radius or the mutual distance between any four points in space is ≤ 3 mm. These points are fractured points to evaluate the stability of the catheter position	Fractured point ratio ≤ 25%	+ 1	+ 1
Gaps	The distance between two points is greater than 6 mm, and there are no other ablation points. This parameter is used to assess the continuity of ablation lesions	Gap ≤ 2	+ 1	+ 1
Ablation Index Strategy	Proportion of ablation points achieving predefined Ablation Index targets: anterior wall ≥ 550, posterior wall ≥ 400, roof ≥ 500	Ablation Index Strategy score ≥ B	+ 1	+ 1
FOT(force-over-time)	Evaluate whether the pressure is above 4 g every 2.5 s in 40% of the time over all lesions in PV ablation, and evaluate whether the ablation pressure is sufficient	FOT(4 g,30%) ≥ 75%	+ 1	+ 1
PVI time	the duration from the first radiofrequency (RF) to the last RF of the Pulmonary veins	the total time of two PVI ≤ 60 min	+ 1	+ 1
PVI +	It is suitable for patients with persistent atrial fibrillation,evaluating additional linear ablation	Number of ablation lines ≥ 2,ablation linear’s FOT(4 g,30%) ≥ 75%, Gap ≤ 0,Ablation Index Strategy ≥ B	+ 1 (if applicable, added after averaging)	

The Vistag score construction and definition of the Parameter. Ablation Index Strategy grading: Grade A: ≥ 90% of target points achieve Ablation Index goal, Grade B: 75–89% achieve Ablation Index goal, Grade C: < 75% achieve Ablation Index goal, Only Grade ≥ B is considered optimal. Left PV score = Fractured points (0/1) + Gap (0/1) + Ablation Index Strategy (0/1) + FOT (0/1) + PVI time (0/1).Right PV score = Fractured points (0/1) + Gap (0/1) + Ablation Index Strategy (0/1) + FOT (0/1) + PVI time (0/1).For patients with persistent atrial fibrillation, an additional PVI + score (maximum + 1 point) is added to the total score if the predefined quality criteria for linear ablation (FOT ≥ 75%, Gap ≤ 0, Ablation Index Strategy ≥ B) are met. Total Vistag Score = [(Left PV score + Right PV score)/2] + PVI + score(0/1),with the result rounded down to the nearest integer (decimal portion discarded)

### Endpoint definition and follow-up strategy

The primary endpoint of this study was recurrence of atrial tachyarrhythmia (including atrial fibrillation, atrial flutter, and atrial tachycardia) during follow-up.

Any documented episode of atrial tachyarrhythmia lasting ≥ 30 s on 12-lead ECG or Holter monitoring after a 3-month blanking period post-ablation.The 3-month blanking period was applied in accordance with current guidelines (2024 ESC Guidelines for the Management of Atrial Fibrillation) to exclude early recurrences related to transient inflammatory effects post-ablation.

Follow-up was conducted at 3, 6, 9 and 12 months post-ablation, including clinical evaluation and 24-h Holter monitoring. Patients were also instructed to undergo additional ECG or Holter monitoring if they experienced symptoms suggestive of arrhythmia between scheduled visits. All enrolled patients completed a minimum follow-up of 365 days.

### Grouping of subjects

The researchers commissioned Johnson & Johnson to export all the Vistag analysis reports of the patients' surgeries. The detailed overall process of this study is presented in Figure 1.

### Statistical analysis

Researchers will enter the research data into EXCEL in detail and accurately, ensuring the consistency of the input data and the original data.SPSS 25.0 statistical software is employed for data analysis. Continuity variables of those conforming to a normal distribution are expressed as mean ± standard deviation (± S). An independent sample t-test is applied for comparison between two different groups. When the distribution does not conform to normality, the median and interquartile range (M(P25, P75) are used to describe the distribution. The Mann–Whitney U test is used for group-to-group comparisons. Categorical data are expressed in terms of frequency and percentage (%), and comparison between groups is performed by the Chi-square test (χ2) or Fisher’s exact test. Recurrence-free survival was estimated using the Kaplan–Meier method, and between-group comparisons were performed using the log-rank test. To identify independent predictors of AF recurrence, multivariate logistic regression analysis was performed, with recurrence treated as a binary outcome (recurrence within 365 days vs. no recurrence). Variables with *P <* 0.05 in baseline data were entered into the multivariate model. Results are reported as odds ratios (OR) with 95% confidence intervals (CI). A two-sided *P <* 0.05 was considered statistically significant.

## Results

### Patient population

From January 2023 to January 2024, a total of 148 patients were enrolled in the study.84 patients were assigned to the high-score group,and the remaining 64 patients who underwent AF ablation during the same period constituted the low-score group. The baseline characteristics of enrolled patients were summarized in Table 2.

**Table 2:** Baseline characteristics of enrolled patients

	The high-score group(*n =* 84)	The low-score group(*n =* 64)	*P*
Age	61.00(54.00,68.00)	66.00(55.25,71.00)	0.816
Height	167.27 ± 8.23	169.13 ± 7.79	0.072
Weight	71.30 ± 14.78	72.19 ± 14.16	0.712
BMI	24.22(21.77,27.76)	23.91(22.63, 26.71)	0.727
FBG	5.80(4.90,6.50)	5.70(5.26, 7.43)	0.395
FTG	1.33(1.03,1.75)	1.03(0.91, 1.32)	0.538
Systolic pressure	129.72 ± 20.25	129.17 ± 17.42	0.496
Diastolic pressure	77.00(70.00,90.00)	82.50(73.25, 88.75)	0.605
BNP	422.00(143.50, 1180.00)	325.00(95.50, 1241.50)	0.749
Left atrium’s size	42.07 ± 6.50	41.00 ± 7.01	0.931
LVEDV	105.37 ± 25.87	112.54 ± 19.63	**0.023**
CRP	0.50(0.50,1.92)	0.62(0.50, 2.96)	0.877
LVEF	63.21 ± 5.21	64.08 ± 6.28	**0.031**
LVESV	36.00(31.00,45.00)	38.00(31.00, 47.50)	**0.041**
Male	54 (64.3%)	41(64.1%)	0.978
SMOKING	20 (23.8%)	21(32.8%)	0.225
Drinking	25 (29.8%)	20(31.3%)	0.845
Hypertension	41 (48.8%)	28(44.4%)	0.600
Diabetes	8 (9.5%)	19(29.5%)	**0.002**
Stroke	10 (12%)	9(14.1%)	0.481
CHD	14(16.7%)	16(25.0%)	0.212
PeAF	49 (58.3%)	35(54.7%)	0.657

Baseline characteristics of enrolled patients

Bold values indicate statistically significant differences between groups (*P <* 0.05)

*FBG* Fasting Blood-Glucose, *FTG* Fasting Triglycerides, *LVEDV* Left Ventricular End—Diastolic Volume, *LVEF* Left Ventricular Ejection Fraction, *LVESV* Left Ventricular End—Systolic Volume, *CHD* Coronary heart disease, *PeAF* Persistent atrial fibrillation,

It reveals no statistically significant differences in age, height, weight, gender distribution, or left atrium’s size between the two groups. However, statistically significant differences were observed in certain baseline parameters, specifically LVESV (Left Ventricular End-Systolic Volume), LVEDV (Left Ventricular End-Diastolic Volume), Left Ventricular Ejection Fraction (LVEF), and diabetes.

### Surgical characteristics and AIFV system characteristics

The surgical characteristics are summarized in Table 3. In the left pulmonary vein antrum, the proportions of patients with FOT ≥ 75%, Fractured points ratio ≤ 25%, and Ablation Index strategy ≥ B ratio were higher in the high-score group than in the low-score group, and a similar trend was observed in the right pulmonary vein.

**Table 3:** Comparison of the AIFV system parameters and procedural outcomes between groups

	The high-score group(*n =* 84)	The low-score group(*n =* 64)	*P*
Surgical time	253.89 ± 63.75	257.86 ± 61.61	0.788
Bilateral PVI Time	59.73 ± 17.67	56.86 ± 16.26	0.279
Left pulmonary vein antrum			
Single PVI Time	30.00(23.00, 35.25)	29.00(23.00, 36.00)	0.585
Fractured points	84(100%)	46(71.9%)	**< 0.001**
FOT	83(98.8%)	48(75.0%)	**< 0.001**
GAPs	84(100%)	63(98.4%)	0.250
Ablation Index strategy	81(96.4%)	38(59.4%)	**< 0.001**
SIPV	65(77.4%)	42 (65.6%)	0.113
Right pulmonary vein antrum			
Single PVI Time	30.50(21.00, 35.25)	25.00(21.00, 34.00)	0.112
Fractured points	82(100%)	52(81.3%)	**< 0.001**
FOT	82(100%)	34(53.1%)	**< 0.001**
GAP	82(100%)	62(98.4%)	0.256
Ablation Index strategy	82(100%)	29 (45.3%)	**< 0.001**
SIPV	52(63.4%)	38(59.4%)	0.618

Surgical characteristics of enrolled patients. Bilateral PVI Time: Bilateral pulmonary vein isolation time; SIPV: Successful Isolation of Pulmonary Vein. Data are presented as n (%) of patients meeting the predefined criteria for each parameter, unless otherwise specified. Bold values indicate statistically significant differences between groups (*P <* 0.05). “*P <* 0.001 “ indicates statistical significance at the 0.1% level

### Follow-up

**Fig. 3:**
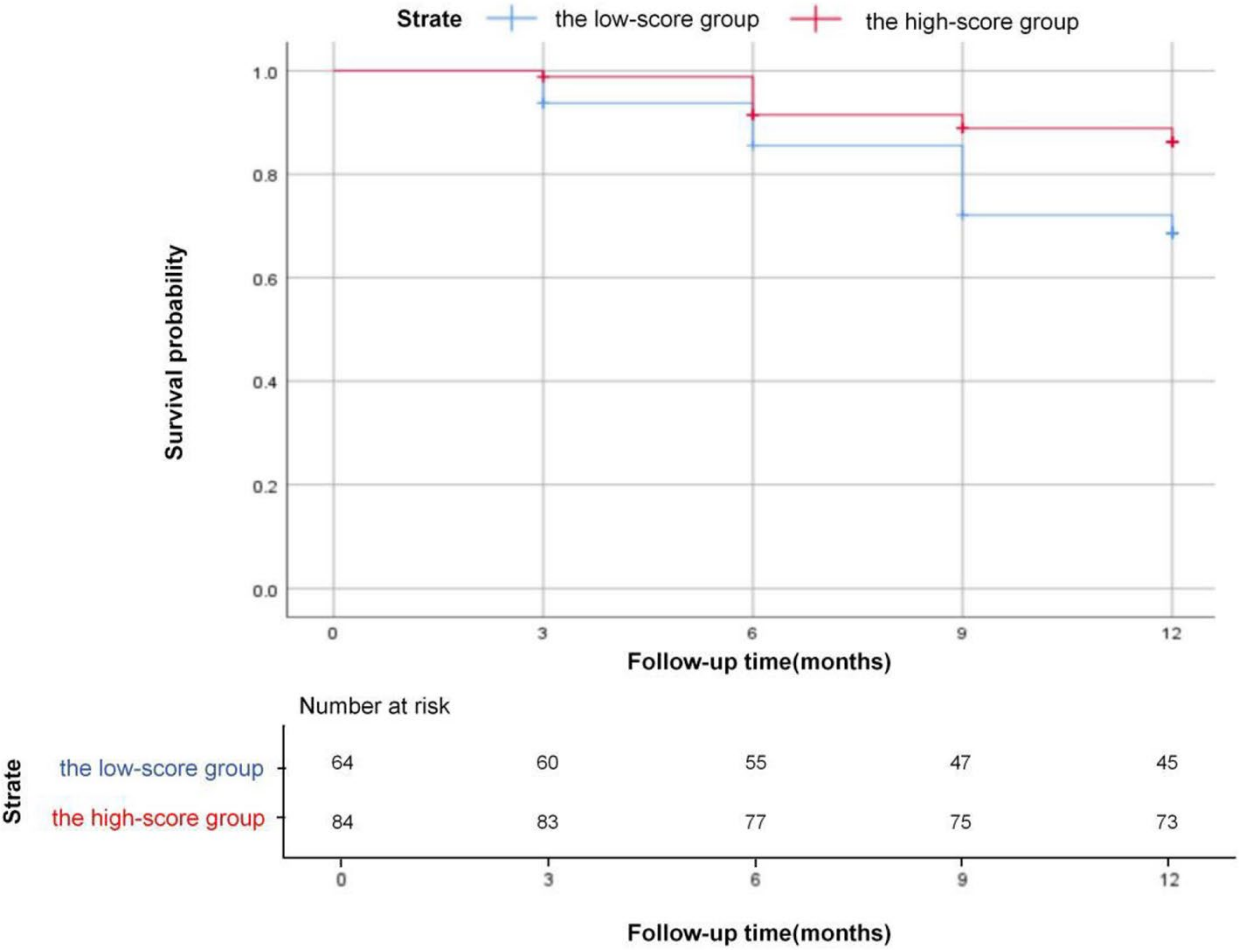
Kaplan–Meier curves for freedom from atrial arrhythmia recurrence after the initial ablation procedure. Number at risk for each group at 0, 3, 6, 9, and 12 months are shown below the figure.The high-score group is shown in red, and the low-score group in blue. Log-rank *P =* 0.012

All patients underwent a minimum of 365 days of follow-up after the initial procedure. In the high-score group, 11 patients experienced recurrence of atrial tachyarrhythmia, as detected by Holter monitoring. In the low-score group, 19 patients experienced recurrences of atrial tachycardia. Kaplan–Meier curves analysis revealed that the atrial arrhythmia-free survival rate was higher in the high-score group than in the low-score group (Fig. 3, log-rank test, *P =* 0.012).

### The AIFV system outcome

**Fig. 4:**
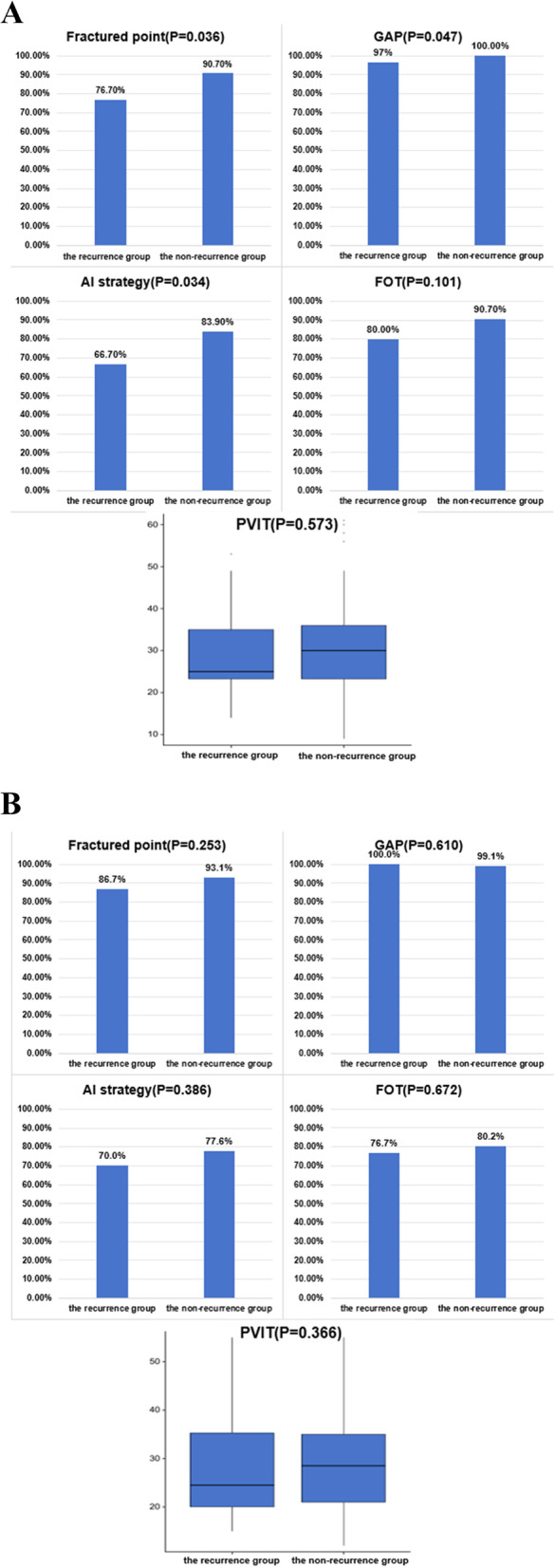
**A** Shows the comparison of the left pulmonary vein between the recurrence group and the non-recurrence group in terms of Fractured points, PVI time, GAPs, Ablation Index strategy, and contact force meeting force over time (FOT). **B** Shows the comparison of the right pulmonary vein between the recurrence group and the non-recurrence group in terms of Fractured points, PVI time, GAPs, Ablation Index strategy, contact force meeting force over time (FOT)

Figure 4A and B, respectively, present the Vistag analysis results of the left and right pulmonary veins between the recurrence group and the non-recurrence group. In the left pulmonary vein, there were significantly more people meeting the criteria of fractured points ≤ 25% (90.7% vs76.7%, *P =* 0.036),Gaps ≤ 2 (100% vs 97%, *P =* 0.047)and Ablation Index strategy ≥ B (83.9%, vs 66.7%, *P =* 0.034) in the non-recurrence group than in the recurrence group. The same trend in the data related to the right pulmonary vein.

### Predictors of AF recurrence

In the multivariate logistic regression analysis, it was shown that the risk of atrial fibrillation recurrence in the high-score group was 0.383 times that of the low-score group (OR = 0.383, 95% CI 0.160–0.912, *P =* 0.030). This indicates that a higher Vistag score is significantly associated with a lower risk of atrial fibrillation recurrence. The detailed results of multivariate logistic regression analyses are reported in Table 4.

**Table 4:** Atrial fibrillation recurrence factors were identified using multivariate logistic regression

	OR	95%CI	*P* value
Diabetes	1.416	0.525–3.818	0.492
LVEF	1.001	0.952–1.051	0.981
The high-score group	0.383	0.160–0.912	**0.030**

Bold values indicate statistically significant differences between groups (*P <* 0.05).All baseline characteristics were first compared between the recurrence and non-recurrence groups using appropriate statistical tests (t-test, Mann–Whitney U test, or chi-square test).Variables with *P <* 0.05 in the univariate baseline comparison were considered for inclusion in the multivariate model.Note on collinearity: LVEDV and LVESV were highly correlated with each other (*r *= 0.82, *P <* 0.001) and with LVEF. To avoid multicollinearity, which can destabilize regression models, we retained only LVEF as the representative echocardiographic variable, as it is the most clinically relevant and widely used measure of ventricular function

**Fig. 5:**
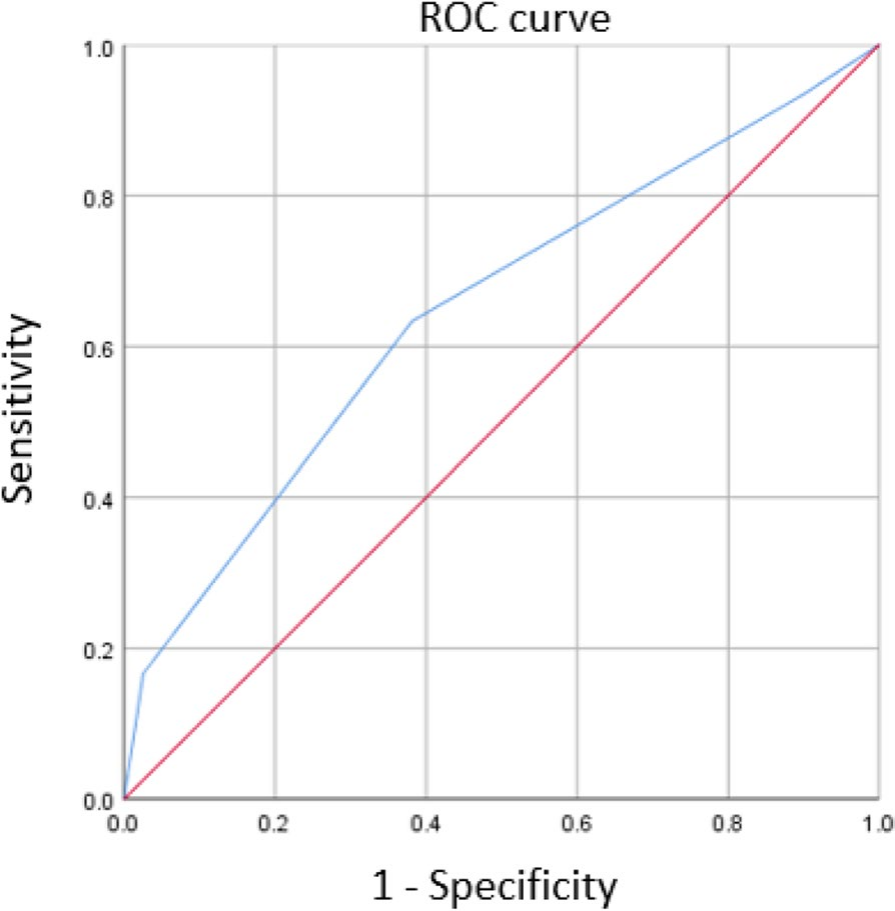
ROC Curve

ROC curve analysis (Fig. 5) for the continuous Vistag score demonstrated moderate discriminative ability for predicting atrial tachyarrhythmia recurrence (AUC = 0.648; 95% CI: 0.531–0.764; *P =* 0.013). The optimal cut-off identified by the Youden index was ≥ 4.5, with a sensitivity of 63.3% and specificity of 61.9%.

Given that the Vistag score is an integer (0–6), a cut-off of ≥ 4.5 is clinically equivalent to the pre-specified threshold of ≥ 5 (i.e., meeting optimal criteria in at least five of six core parameters). This concordance supports the clinical validity of the pre-specified grouping strategy.

The modest AUC reflects that the Vistag score, as a composite quality metric, is not intended as a standalone risk predictor. Its prognostic value is best appreciated after multivariable adjustment, where the pre-specified threshold of ≥ 5 remained independently associated with reduced recurrence risk (OR = 0.383, 95% CI 0.160–0.912, *P =* 0.030).

## Discussion

Our study demonstrates that a higher Vistag score on the AIFV System is associated with a significantly lower risk of atrial fibrillation recurrence, supporting our initial hypothesis. The Vistag analysis report serves as a multidimensional assessment tool, integrating several critical procedural parameters, including catheter stability, ablation continuity, contact force sustainability, ablation index, and procedural duration. These parameters collectively influence ablation efficacy. In the following sections, we will discuss the implications of our findings in relation to each key parameter and the existing literature.

Catheter stability and ablation safety are reflected in the “Fractured points” metric, which assesses the spatial distribution of ablation tags. A lower proportion of fractured points (≤ 25%) was significantly more prevalent in the non-recurrence group. This suggests that stable catheter positioning during ablation may reduce unnecessary tissue injury and enhance lesion uniformity. This aligns with the principle that consistent catheter contact facilitates transmural lesion formation and reduces the likelihood of gap formation [24, 25].

Ablation continuity, evaluated by the “Gaps” parameter, is crucial for achieving durable pulmonary vein isolation (PVI). Our data show that the high-score group had a higher proportion of cases meeting the gaps ≤ 2 criterion, indicating more contiguous lesion sets. Incomplete ablation lines are a well-known cause of electrical reconnection and AF recurrence [24–28]. Over time, as the acute effects of radiofrequency energy diminish, the transient tissue damage induced by exponential ablation gradually recovers, resulting in the formation of conduction gaps. These gaps may allow pulmonary vein (PV) triggers to activate the adjacent left atrium, thereby initiating atrial fibrillation (AF) [29, 30]. Thus, a systematic assessment of ablation continuity may help operators optimize lesion deployment in real-time.

Contact force sustainability, measured by FOT (force-over-time), was significantly higher in the non-recurrence group. Adequate and maintained contact force is essential for effective lesion depth and transmurality [31, 32]. Insufficient force may lead to suboptimal lesion formation and subsequent recovery of conduction, ultimately contributing to recurrence [29, 30]. The AIFV system’s FOT metric provides an objective measure of contact force adequacy, which may guide intraoperative adjustments.

Ablation index strategy ≥ B was more frequently achieved in the non-recurrence group. Ablation Index integrates contact force, time, and power to estimate lesion quality, and its prospective use has been shown to improve clinical outcomes after ablation of atrial fibrillation [33]. Our findings further support the utility of ablation index-guided ablation in achieving durable PVI.

An extended ablation strategy, such as linear ablation or ethanol infusion of the vein of Marshall (EIVOM), was assessed through the PVI + module. Although not all patients received adjunctive ablation, the system’s ability to evaluate linear lesion quality may help stratify recurrence risk in more complex cases, consistent with recent evidence [22].

Several factors may explain the lack of significant differences in right pulmonary vein parameters between patients with and without AF recurrence. First, anatomical and electrophysiological differences between left and right PVs may render left-sided parameters more determinative of clinical outcomes [34, 35]. Second, technical factors may make right PV isolation more consistently achievable. Third, the high achievement rates for right PV parameters (e.g., Gap ≤ 2 in > 98% of patients) reduce statistical variability.

This result does not align with the findings of Dennis W. den Uijl et al., who previously demonstrated the impact of left atrial fibrosis and left atrial dimensions on the outcomes of catheter ablation in patients with atrial fibrillation [36]. Additionally, Jianhui Zhuang et al. demonstrated that left atrium’s size is associated with an increased risk of atrial fibrillation recurrence following single-catheter pulmonary vein isolation [37].

The quality of ablation surgery has a direct impact on the risk of atrial fibrillation recurrence. Therefore, evaluating the effectiveness of ablation procedures is crucial [38]. Using the AIFV system, this study investigated the relationship between the quality of radiofrequency catheter ablation and the recurrence of atrial fibrillation.

The AIFV system offers several key advantages. First, it provides an objective and reproducible assessment that eliminates operator-dependent variability in evaluating procedural quality. Second, it enables multidimensional integration, capturing the full complexity of ablation quality across multiple complementary parameters. Third, it delivers independent prognostic value, improving risk stratification beyond traditional clinical factors. Fourth, it facilitates standardization across centers and operators, supporting quality benchmarking and enabling multi-center research collaborations.

## Limitations

This was a single-center study with a modest sample size and a follow-up period of one year. The AIFV system itself is dependent on the accuracy of CARTO3 data export and artificial intelligence interpretation, which may introduce technical variability. Future multi-center, larger-scale studies with longer follow-up are needed to validate our findings.Several limitations should be acknowledged regarding rhythm monitoring. The use of intermittent 24-h Holter monitoring (at 3, 6, 9 and 12 months) and symptom-driven ECGs, rather than continuous rhythm monitoring (e.g., with implantable loop recorders), may have underestimated the true incidence of asymptomatic or paroxysmal recurrences occurring between scheduled visits.

## Conclusions

Using the AIFV system, this study demonstrates that the Vistag score is significantly associated with postoperative atrial fibrillation recurrence, with higher scores predicting lower risk. This association underscores the critical role of procedural precision in catheter ablation outcomes.

The AIFV system offers two principal added values in AF ablation. First, it enables objective quantification of ablation quality. Conventional assessment relies heavily on operator experience and subjective visual interpretation of electroanatomical maps. By transforming raw CARTO3 data into standardized, reproducible Vistag metrics, the AIFV system reduces inter-operator variability and enables consistent quality benchmarking across procedures and institutions. Second, the system demonstrates independent prognostic capability. The Vistag score remained significantly associated with recurrence risk after multivariable adjustment, providing a novel, data-driven tool for postoperative risk stratification. This prognostic information may guide follow-up intensity, inform patient counseling, and identify candidates for early re-intervention.

Collectively, these added values position the AIFV system not only as a quality assessment tool but also as a predictive decision-support platform for the precision management of atrial fibrillation.

## Data Availability

The datasets used during the current study are available from the corresponding author on reasonable request.

## Abbreviations

AF: Atrial Fibrillation
AIFV System: An artificial intelligence-based ablation quality analysis system that generates structured Vistag scores from CARTO3 mapping data
BNP: B-type Natriuretic Peptide
BMI: Body Mass Index
B-PVIT: Bilateral Pulmonary Vein Isolation Time
CHD: Coronary Heart Disease
CI: Confidence Interval
CRP: C-reactive Protein
DCCV: Direct-Current Cardioversion
EIVOM: Ethanol Infusion of the Vein of Marshall
FBG: Fasting Blood-Glucose
FOT: Force-Over-Time
FTG: Fasting Triglycerides
LVEDV: Left Ventricular End-Diastolic Volume
LVEF: Left Ventricular Ejection Fraction
LVESV: Left Ventricular End-Systolic Volume
OR: Odds Ratio
PeAF: Persistent Atrial Fibrillation
PV: Pulmonary Vein
PVI: Pulmonary Vein Isolation
PVI +: Extended Ablation Strategy (e.g., beyond PVI)
RF: Radiofrequency
RFCA: Radiofrequency Catheter Ablation
SIPV: Successful Isolation of Pulmonary Vein
